# The risk of bias in randomized controlled trials in otorhinolaryngology: hardly any improvement since 1950

**DOI:** 10.1186/s12901-017-0036-x

**Published:** 2017-04-18

**Authors:** Jeroen P. M. Peters, Inge Stegeman, Wilko Grolman, Lotty Hooft

**Affiliations:** 1 Department of Otorhinolaryngology and Head & Neck Surgery, University Medical Center Utrecht, House Postal Number G05.129, PO BOX 85500, 3508 GA Utrecht, The Netherlands; 2 Brain Center Rudolf Magnus, University Medical Center Utrecht, Utrecht, The Netherlands; 3 Cochrane Netherlands, Julius Center for Health Sciences and Primary Care, University Medical Center Utrecht, Utrecht, The Netherlands

**Keywords:** Risk of Bias, Bias, Otorhinolaryngology, Randomized Controlled Trial, Cochrane, Quality of reporting

## Abstract

**Background:**

Randomized Controlled Trials (RCTs) represent the most valuable study design to evaluate the effectiveness of therapeutic interventions. However, flaws in design, conduct, analysis, and reporting of RCTs can cause the effect of an intervention to be under- or overestimated. These biased RCTs may be included in literature reviews. To make the assessment of Risk of Bias (RoB) consistent and transparent, Cochrane published a RoB tool, with which RoB is assessed per item as “low”, “unclear” or “high”. Our objective was to provide an overview of RoB assessments of RCTs in otorhinolaryngology over time, and to identify items where improvement is still warranted.

**Methods:**

We retrieved Cochrane reviews in the otorhinolaryngologic research field published in 2012 and 2013. We used all judgments per item as assessed by the review authors of the included RCTs. We evaluated the association between “low RoB” vs. “unclear and high RoB” and the year of publication (time strata: ‘<1990’, ‘1990–1995’, ‘1996–2000’, ‘2001–2005’, ‘2006–2012’) per item using binary logistic regression.

**Results:**

We extracted the RoB assessments from 42 Cochrane reviews that had included 402 RCTs (median number of RCTs per review: 7, range 1–40). In total 2,356 items were assessed (mean number of assessed items per RCT: 5.9, standard deviation 1.8). On binary logistic regression, RCTs published in 2006–2012, compared with those published before 1990, were more likely to have a low RoB for two items: *random sequence generation* (odds ratio 6.09 [95% confidence interval: 3.11–11.95]) and *allocation concealment* (3.59 [1.87–6.90]). On all other items, there was no significant increase in the proportion of low RoB when comparing RCTs published in 2006–2012 with RCTs published before 1990.

**Conclusion:**

Although there were some positive developments in the RoB assessments in otorhinolaryngology, a further decrease in RoB is still warranted on several items. Currently, biased RCTs are included in Cochrane reviews and effects of therapeutic interventions can be under- or overestimated, with implications for clinical patient care.

**Electronic supplementary material:**

The online version of this article (doi:10.1186/s12901-017-0036-x) contains supplementary material, which is available to authorized users.

## Background

Randomized Controlled Trials (RCTs) represent the most valuable study design for individual studies to evaluate the effectiveness of therapeutic interventions. Adequate randomization ensures that known and unknown confounding factors are distributed evenly across groups. However, flaws in the design, conduct, analysis, and reporting of RCTs can cause the effect of an intervention to be under- or overestimated [1, 2]; this is referred to as “bias”. When biased RCTs are included in literature reviews, the findings of these reviews may also be biased [3, 4]. Since the conclusions of reviews are used directly in clinical practice, patients may be at risk because of poorly conducted RCTs.

To make the process of assessing Risk of Bias (RoB) more consistent and transparent, Cochrane developed and validated the Cochrane RoB tool. The first version of the Cochrane RoB tool was presented in 2008 [5], and in 2011 a revision was published [1] (the most recent version can be accessed online [6]). In the revised version, separate assessments were recommended for some items (e.g. not only assess *blinding*, but assess *blinding of participants and personnel* and *blinding of outcome assessment* separately). Cochrane recommends authors of Cochrane reviews to carefully consider the potential limitations of the included studies to obtain reliable conclusions [1] and to discuss the impact of including trials with a high RoB on the results of the Cochrane review. Using the Cochrane RoB tool, authors of Cochrane reviews classify the RoB in the included RCT on specific items in three categories (“low”, “unclear” or “high” RoB). In the Cochrane RoB tool, the following items are included: 1) selection bias (items: *random sequence generation*, *allocation concealment*), 2) performance bias (*blinding of participants and personnel*), 3) detection bias (*blinding of outcome assessment*), 4) attrition bias (*incomplete outcome data*), 5) reporting bias (*selective reporting*), and 6) other bias (*other sources of bias*).

In this paper, we provide an overview of the RoB assessments in the literature of the otorhinolaryngologic research field. We aimed to assess how the RoB has developed over time per item. We hypothesize that the RoB has decreased over time for all items. Subsequently, we identified items where improvement is still warranted.

## Methods

### Selection of Cochrane reviews

We retrieved all Cochrane reviews on otorhinolaryngologic topics published in 2012 and 2013. The search syntax to retrieve these reviews in PubMed was described in detail elsewhere [7], and also uploaded as Additional file 1. In short, search syntaxes for otorhinolaryngologic articles [8], reviews [9] and the journal (*Cochrane Database of Systematic Reviews*) were used and restricted for publication type (no editorials, letters to the editor, news or comments) and publication date (2012 and 2013).

Two authors (JPMP and IS) assessed whether the retrieved Cochrane reviews were truly conducted in otorhinolaryngology.

### RoB assessments

We collected the year of publication, the total number of included RCTs, and the RoB assessments (“low”, “unclear” or “high” RoB) per item as judged by the original review authors from the included reviews.

All Cochrane review authors used the Cochrane RoB tool [6]. However, some review authors did not use all items or used sub-categories for certain items. For example, sometimes *blinding* was assessed, whereas newer reviews assessed *blinding of participants and personnel* and *blinding of outcome assessments* separately. When multiple outcomes were assessed, we adopted the RoB assessment of the primary reported outcome.

### Data analysis

Descriptive statistics of the included Cochrane reviews and of the RoB assessments of the individual RCTs were computed. The frequency of low, unclear and high RoB was calculated per item. Subsequently, we calculated the proportion of items that were scored as low, unclear or high RoB per item per time stratum. Therefore, we divided all RCTs in five time strata based on the year of publication: ‘<1990’, ‘1990–1995’, ‘1996–2000’, ‘2001–2005’, and ‘2006–2012’.

To explore the development of RoB over time, we performed a binary logistic regression analysis. The RoB assessment “low” was the reference category, and was compared to “unclear and high” RoB per item; this resulted in an odds ratio (OR, with 95% confidence intervals (CI)) per time stratum with ‘<1990’ as reference time stratum.

Statistical package SPSS v22 was used. A *p*-value of < .05 was considered statistically significant.

## Results

### Selection of Cochrane reviews

**Fig. 1:**
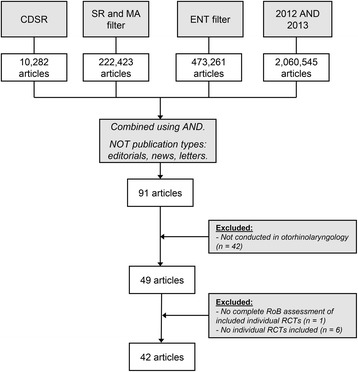
Flowchart. Date of search: September 3, 2014 [7]. For full syntax, see Additional file 1

The search retrieved 91 Cochrane reviews, of which 42 reviews were not conducted in the otorhinolaryngologic research field. The remaining 49 Cochrane reviews were included in our study (Fig. 1). Of these 49 articles, six did not include any RCTs (so called “empty reviews”) and thus did not assess RoB. Furthermore, one review only reported individual items that had high RoB in the included RCTs, but did not report on items that might have had a low or unclear RoB. Consequently, these seven reviews were excluded leaving a total of 42 included reviews (22 were published in 2012, 20 were published in 2013).

### Selection of individual RCTs

All 42 reviews included a total of 402 individual RCTs (median number of RCTs included per review: 7, range 1–40). The median year of publication of the individual RCTs was 1998 (range 1950–2012). We included different, but comparable, numbers of RCTs per time stratum (<1990: *n* = 96, 1990–1995: *n* = 66, 1996–2000: *n* = 69, 2001–2005: *n* = 92, and 2006–2012: *n* = 79).

Of the 402 included RCTs, 10 were reported in two reviews; thus, 392 unique RCTs were assessed. Since the number of overlapping RCTs is so little (<2.5%), we based our analysis on the 402 RoB assessments. Moreover, the RoB assessments of the overlapping RCTs were often different between reviews (data not shown).

### RoB assessments

In total, 2,356 items were assessed (mean number of items per RCT 5.9, standard deviation 1.8).

As explained before, review authors used different versions of the Cochrane RoB tool, which resulted in a different total number of assessment per item. All items of the standard Cochrane RoB tool [6] were assessed more than 100 times: *random sequence generation* (*n* = 384), *allocation concealment* (*n* = 399), *blinding of participants and personnel* (*n* = 137), *blinding of outcome assessment* (*n* = 130), *blinding* (*n* = 227), *incomplete outcome data* (*n* = 345), *selective reporting* (*n* = 337) and *other bias* (*n* = 273). All other items were assessed <20 times (Additional file 2). For readability of the remainder of this paper, in our following analyses we only assessed the items from the standard Cochrane RoB tool [6]. Together, these items constituted 2,232 (94.7%) of all 2,356 RoB assessments and therefore form a representative sample of our data. Thirty-six studies (9.0%) had a low RoB on all assessed items, and 208 studies (51.7%) had at least one item with a high RoB.

**Fig. 2:**
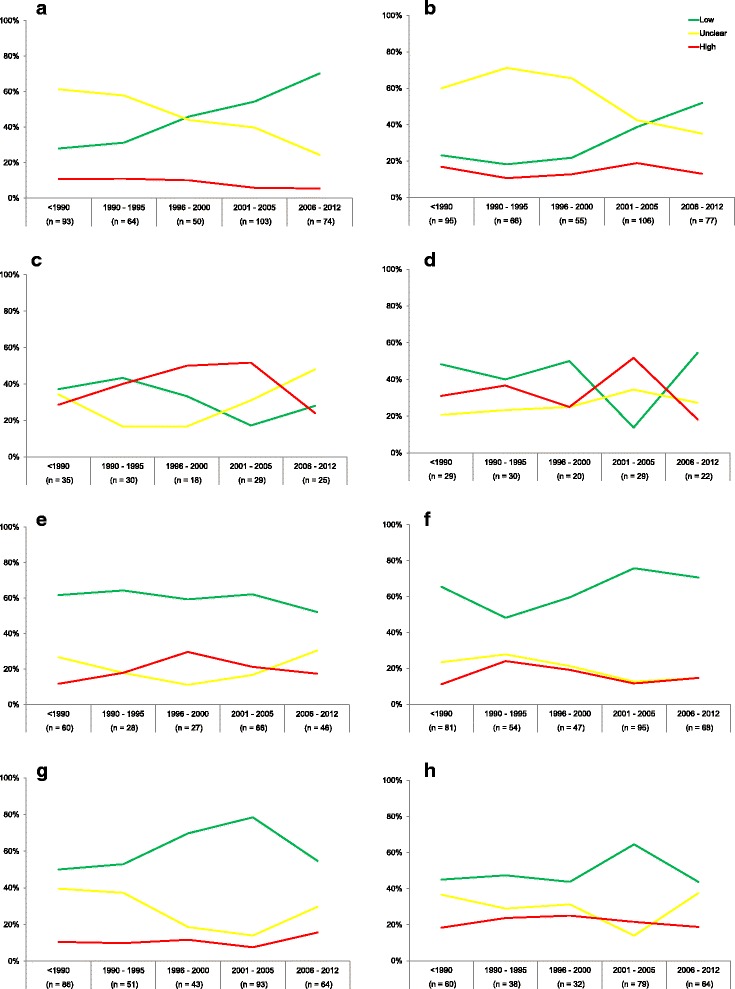
**a-h** Development of RoB per item per time stratum. **a**) random sequence generation, **b**) allocation concealment, **c**) blinding of participants and personnel, **d**) blinding of outcome assessment, **e**) blinding, **f**) selective reporting, **g**) incomplete outcome data, and **h**) other bias. X-axis: time strata (number of assessments). Y-axis: proportion of articles that had low (*green line*), unclear (*yellow line*) or high (*red line*) RoB

Figures 2a-h show the proportions of low, unclear and high RoB per item per time stratum (for the data tables of these figures, see Additional file 3). For example in Fig. 2a the development of RoB over time for *random sequence generation* is depicted. Before 1990, 28% of RCTs had a low RoB for *random sequence generation*, whereas the proportion of RCTs with a low RoB was 70% for RCTs published between 2006–2012. Between these two time strata, a gradual increase in the proportion of low RoB can be observed. The proportion of items that were assessed as unclear RoB gradually declined from 61% (<1990) to 24% (2006–2012), and the proportion of items that were assessed as high RoB slightly declined (<1990: 11%, 2006–2012: 5%). For other items, the development over time is less gradually (e.g. *blinding outcome assessment*).

When we look at the data for *random sequence generation* using binary logistic regression (Table 1), we note that there was no statistically significant difference in the odds for low RoB between RCTs published in 1990–1995 compared to RCTs published before 1990. However, RCTs that were published in all three later time strata were significantly more likely to score a low RoB compared to RCTs published before 1990 (1996–2000: 2.20 (1.17–4.50), 2001–2005: 3.07 (1.69–5.57), 2006–2012: 6.09 (3.11–11.95)).

**Table 1:** Binary logistic regression analysis

Time strata	Random sequence generation (*n* = 384)	Allocation concealment (*n* = 399)	Blinding participants and personnel (*n* = 137)	Blinding outcome assessment (*n* = 130)	Blinding (*n* = 227)	Incomplete outcome data (*n* = 345)	Selective reporting (*n* = 337)	Other bias (*n* = 273)
<1990	1.00	1.00	1.00	1.00	1.00	1.00	1.00	1.00
1990–1995	1.17 (0.58–2.35)	0.74 (0.34–1.62)	1.29 (0.48–3.50)	0.71 (0.26–2.00)	1.12 (0.44–2.84)	**0.49 (0.24–0.99)**	1.13 (0.56–2.26)	1.10 (0.49–2.49)
1996–2000	**2.20 (1.07–4.50)**	0.93 (0.42–2.06)	0.85 (0.26–2.80)	1.07 (0.34–3.35)	0.90 (0.36–2.29)	0.78 (0.37–1.63)	**2.31 (1.06–5.01)**	0.95 (0.40–2.26)
2001–2005	**3.07 (1.69–5.57)**	**2.09 (1.13–3.88)**	0.35 (0.11–1.15)	**0.17 (0.05–0.62)**	1.019 (0.50–2.09)	1.65 (0.86–3.19)	**3.65 (1.90–7.00)**	**2.23 (1.12–4.42)**
2006–2012	**6.09 (3.11–11.95)**	**3.59 (1.87–6.90)**	0.66 (0.22–2.00)	0.93 (0.42–3.91)	0.68 (0.31–1.48)	1.27 (0.63–2.54)	1.21 (0.63–2.31)	0.95 (0.49–1.93)

Legend: The odds ratios for scoring a low RoB compared to RCTs published in the time stratum ‘<1990’ are presented per time stratum per item. We used RoB assessment “low” as the reference category in a binary logistic regression analysis in comparison with RoB assessments “unclear” and “high” taken together, and time stratum ‘<1990’ was the reference time stratum. Values in bold font are statistically significant (*p* < .05)

Also for *allocation concealment*, RCTs that were published in the two latest time strata were significantly more likely to score a low RoB than RCTs published before 1990 (2001–2005: 2.09 (1.13–3.88), 2006–2012: 3.59 (1.87–6.90)). For the items *selective reporting* and *other bias*, RCTs that were published in later time strata had significantly lower RoB than RCTs published before 1990 (selective reporting*:* 1996–2000: 2.31 (1.06–5.01), 2001–2005: 3.65 (1.90–7.00); other bias: 2001–2005: 2.23 (1.12–4.42)).

On the other hand, for the items *blinding outcome assessment* and *incomplete outcome data*, RCTs that were published in later time strata were significantly more likely to have an unclear or high RoB than RCTs published before 1990 (blinding outcome assessment*:* 2001–2005: 0.17 (0.05–0.62); incomplete outcome data: 1990–1995: 0.49 (0.24–0.99)).

## Discussion

We provided an overview of the development of RoB in a sample of otorhinolaryngologic RCTs published from 1950–2012. When looking per item, *random sequence generation* and *allocation concealment* were significantly more likely to score a low RoB when comparing RCTs published between 2006–2012 to RCTs published before 1990. These two items are two of the key factors that make RCTs the most valuable study design to evaluate the effectiveness of therapeutic interventions, so we consider this a positive development.

### Comparison with literature

An analysis like ours was performed by Reveiz et al. [11], who analyzed all RoB assessments in issue 12 (2012) of the *Cochrane Database of Systematic Reviews*; they thus investigated multiple medical specialties. They identified a lower RoB on items *random sequence generation*, *allocation concealment*, *incomplete outcome data* and *selective reporting* for articles published between 2006–2012 compared to articles published before 1990. These data are concordant with our findings with respect to items *random sequence generation* and *allocation concealment*.

They found that the rate of RCTs judged as having a low or high RoB significantly increased over time, whereas the rate of RCTs judged as having an unclear RoB decreased for several domains. In our study, we also observed that the rate of RCTs judged as having an unclear RoB decreased over time for items *random sequence generation* and *allocation concealment*. This would reflect better reporting of items, since the review authors could adequately identify the RoB from the articles. On the other hand, in our sample for items *blinding of participants and personnel* and *blinding*, the rate of RCTs judged as having an unclear RoB increased over time. In the RCTs published between 2006–2012, the proportion of RCTs with an unclear RoB was 48% and 30%, respectively (Additional file 3). We think these proportions are very large, and could easily decrease if authors reported their RCTs better. To help authors report their RCTs better, the Consolidated Standards of Reporting Trials (CONSORT) Statement (www.consort-statement.org) was developed [12] (and later revised in 2001 [10] and 2010 [13, 14]). The CONSORT Checklist lists all important items that should be reported in an RCT. Adherence to the CONSORT reporting guideline has been associated with improved reporting [15, 16].

Yordanov et al. also published an analysis of RoB similar to ours, based on 1,286 trials from multiple medical specialties [17]. They also identified that blinding was not often done properly in many included trials. Additionally, they calculated the avoidable waste of research, as identified previously by Chalmers and Glasziou [18]: easy methodological adjustments at no or little cost were possible to lower the RoB in 50% of trials [17].

Both articles included RCTs from multiple medical specialties. A medical specialty-specific analysis like ours has not been performed previously; hence, we cannot directly compare our findings to specific other medical specialties.

### Methodological considerations

Our study is characterized by several strengths. We based our conclusions on a large sample (*n* = 2,356 RoB assessments in 402 RCTs) of otorhinolaryngologic literature. Furthermore, we used a transparent strategy to yield our final selection of studies. Finally, we performed a unique analysis in our research field and we hope to inspire research groups from other medical disciplines to conduct a similar analysis.

However, we must also take some uncertainties of our study into account. First, our sample of Cochrane reviews and RCTs may be biased: not all therapeutic interventions in otorhinolaryngology have been researched in a randomized study design (e.g. surgical interventions for which randomization is considered unethical), nor have they been reviewed in a Cochrane review. Therefore, some subspecialties may be underrepresented in our sample, limiting the generalizability of our findings to the total field of otorhinolaryngology. Second, we did not look into the specific types of RCTs in our analysis. One could argue that RCTs with a placebo-controlled design may have a lower RoB, and studies with a pragmatic study design may have a higher RoB (because the determinant is not standardized). However, there were only three pragmatic trials in our sample; a subanalysis for these articles would not have been feasible, or have a significant impact on our findings. Third, the RoB assessments were done by individual Cochrane review authors and may have been done inconsistently across the reviews. However, all Cochrane Centres provide training and support for all Cochrane authors on “*Writing a Cochrane review of intervention studies*” to increase their skills and knowledge (including how to assess RoB). Therefore, we assume that all Cochrane authors have assessed the RoB of the included RCTs based on the Cochrane Handbook [6] (minimum two independent reviewers, consensus must be reached, etc.). Although even these assessments remain subjective, this is the best possible standardization of RoB assessments. Finally, there is limited possibility to improve the high RoB identified in items *blinding of participants and personnel*, *blinding of outcome assessment*, and *blinding* (Additional file 3). We acknowledge that blinding may be difficult to perform in otorhinolaryngology, as investigated treatments are often surgical interventions [19]. However, authors should then report that they were unable to blind patients or outcome assessors due to the nature of the investigated intervention, so that the RoB is clear for readers and review authors.

### Recommendation

In our analysis, we observed that two key items of RCTs (*random sequence generation* and *allocation concealment*) improved over time. However, the other six items did not show significant improvement over time. Of these six items, three items were associated with blinding; RoB on these items can never be completely eliminated in surgical trials. In contrast, researchers should focus on proper design, conduct, and reports of RCTs for the other three items (*incomplete outcome data*, *selective reporting* and *other bias*).

Furthermore, for all these six items, the proportion of unclear RoB can be decreased by transparent reporting of RCTs. Researchers may find the CONSORT Statement helpful to check if all important items have been addressed in their manuscript [13, 14]. Finally, we encourage journal editors to strictly adhere to reporting guidelines, and embed the reporting guidelines in their submission process. Ultimately, all these actions will lead to increased value of research findings and to higher quality of patient care.

## Conclusion

We provided an overview of the development of RoB in a selected sample of otorhinolaryngological RCTs published from 1950–2012. When looking at specific items, *random sequence generation* and *allocation concealment* were significantly more likely to score a low RoB when comparing RCTs published between 2006–2012 to RCTs published before 1990. On all other items, there was no significant increase in the proportion of low RoB when comparing RCTs published in 2006–2012 with RCTs published before 1990.

## Additional files

Additional file 1: Search strategy. Date of search: 3 September 2014 [7]. (DOCX 23 kb)

Additional file 2: Number of assessments per item. Items 1–8 are sorted in order of appearance in Cochrane’s RoB tool [6]. Items 9–18 are sorted in order of total number of assessments. Items 9–18 are left out of the analysis (see manuscript). (DOCX 17 kb)

Additional file 3: Data table of Fig. 2a-h. Data table with data of Fig. 2a-h: Development of RoB per item per time stratum. (DOCX 23 kb)

## Abbreviations

CI: Confidence interval
CONSORT Statement: Consolidated standards of reporting trials statement
RCT: Randomized Controlled Trial
RoB: Risk of Bias
