# Supplementary material for: The risk of bias in randomized controlled trials in otorhinolaryngology: hardly any improvement since 1950
Source: BMC Ear Nose Throat Disord. 2017 Apr 18;17:3. doi: 10.1186/s12901-017-0036-x (PMC5395869; doi:10.1186/s12901-017-0036-x)
Supplement: Supplementary file 2 — Number of assessments per item. Items 1–8 are sorted in order of appearance in Cochrane’s RoB tool [6]. Items 9–18 are sorted in order of total number of assessments. Items 9–18 are left out of the analysis (see manuscript). (DOCX 17 kb) [file 12901_2017_36_MOESM2_ESM.docx]

**ADDITIONAL FILE 2: number of assessments per RoB item**

| **RoB item** | | **Total number of assessments** |
| --- | --- | --- |
| 1 | Random sequence generation (selection bias) | 384 |
| 2 | Allocation concealment (selection bias) | 399 |
| 3 | Blinding participants personnel (performance bias) | 137 |
| 4 | Blinding outcome assessment (detection bias) | 130 |
| 5 | Blinding (performance bias and detection bias) | 227 |
| 6 | Incomplete outcome data (attrition bias) | 345 |
| 7 | Selective reporting (reporting bias) | 337 |
| 8 | Other bias | 273 |
|  | | |
| 9 | Blinding participants and personnel 2^nd^ outcome | 15 |
| 10 | Blinding participants personnel 3^rd^ outcome | 15 |
| 11 | Blinding outcome assessment 2^nd^ outcome | 15 |
| 12 | Blinding outcome assessment 3^rd^ outcome | 15 |
| 13 | Baseline imbalances | 15 |
| 14 | Blinding participants | 15 |
| 15 | Blinding assessors | 15 |
| 16 | Comparability of groups at pre-test | 8 |
| 17 | Validated outcome assessments | 7 |
| 18 | Follow up | 2 |
| 19 | Certainty of diagnosis | 2 |

**Legend:**

Items 1-8 are sorted in order of appearance in Cochrane Collaboration’s RoB tool [6]. Items 9-18 are sorted in order of total number of assessments. Items 9-18 are left out of the analysis.
