# Supplementary material for: The risk of bias in randomized controlled trials in otorhinolaryngology: hardly any improvement since 1950
Source: BMC Ear Nose Throat Disord. 2017 Apr 18;17:3. doi: 10.1186/s12901-017-0036-x (PMC5395869; doi:10.1186/s12901-017-0036-x)
Supplement: Supplementary file 3 — Data table of Fig. 2a-h. Data table with data of Fig. 2a-h: Development of RoB per item per time stratum. (DOCX 23 kb) [file 12901_2017_36_MOESM3_ESM.docx]

**ADDITIONAL FILE 3: Data of Figure 2a-h**

| **Time period** | **RoB judgment** | **Random sequence generation**  **(n = 384)** | **Allocation concealment (n = 399)** | **Blinding participants and personnel**  **(n = 137)** | **Blinding outcome assessment**  **(n = 130)** | **Blinding (n = 227)** | **Incomplete outcome data (n = 345)** | **Selective reporting (n = 337)** | **Other bias (n = 273)** |
| --- | --- | --- | --- | --- | --- | --- | --- | --- | --- |
| <1990 | Low | 26 (28%) | 22 (23%) | 13 (37%) | 14 (48%) | 37 (62%) | 53 (65%) | 43 (50%) | 27 (45%) |
|  | Unclear | 57 (61%) | 57 (60%) | 12 (34%) | 6 (21%) | 16 (27%) | 19 (23%) | 34 (40%) | 22 (37%) |
|  | High | 10 (11%) | 16 (17%) | 10 (29%) | 9 (31%) | 7 (12%) | 9 (11%) | 9 (10%) | 11 (18%) |
|  | **Total** | **93 (100%)** | **95 (100%)** | **35 (100%)** | **29 (100%)** | **60 (100%)** | **81 (100%)** | **86 (100%)** | **60 (100%)** |
|  | | | | | | | | | |
| 1990 – 1995 | Low | 20 (31%) | 12 (18%) | 13 (43%) | 12 (40%) | 18 (64%) | 26 (48%) | 27 (53%) | 18 (47%) |
|  | Unclear | 37 (58%) | 47 (71%) | 5 (17%) | 7 (23%) | 5 (18%) | 15 (28%) | 19 (37%) | 11 (29%) |
|  | High | 7 (11%) | 7 (11%) | 12 (40%) | 11 (37%) | 5 (18%) | 13 (24%) | 5 (10%) | 9 (24%) |
|  | **Total** | **64 (100%)** | **66 (100%)** | **30 (100%)** | **30 (100%)** | **28 (100%)** | **54 (100%)** | **51 (100%)** | **38 (100%)** |
|  | | | | | | | | | |
| 1996 – 2000 | Low | 23 (46%) | 12 (22%) | 6 (33%) | 10 (50%) | 16 (59%) | 28 (60%) | 30 (70%) | 14 (44%) |
|  | Unclear | 22 (44%) | 36 (65%) | 3 (17%) | 5 (25%) | 3 (11%) | 10 (21%) | 8 (19%) | 10 (31%) |
|  | High | 5 (10%) | 7 (13%) | 9 (50%) | 5 (25%) | 8 (30%) | 9 (19%) | 5 (12%) | 8 (25%) |
|  | **Total** | **50 (100%)** | **55 (100%)** | **18 (100%)** | **20 (100%)** | **27 (100%)** | **47 (100%)** | **43 (100%)** | **32 (100%)** |
|  | | | | | | | | | |
| 2001 – 2005 | Low | 56 (54%) | 41 (39%) | 5 (17%) | 4 (14%) | 41 (62%) | 72 (76%) | 73 (78%) | 51 (65%) |
|  | Unclear | 41 (40%) | 45 (42%) | 9 (31%) | 10 (34%) | 11 (17%) | 12 (13%) | 13 (14%) | 11 (14%) |
|  | High | 6 (6%) | 20 (19%) | 15 (52%) | 15 (52%) | 14 (21%) | 11 (12%) | 7 (8%) | 17 (22%) |
|  | **Total** | **103 (100%)** | **106 (100%)** | **29 (100%)** | **29 (100%)** | **66 (100%)** | **95 (100%)** | **93 (100%)** | **79 (100%)** |
|  | | | | | | | | | |
| 2006 – 2012 | Low | 52 (70%) | 40 (52%) | 7 (28%) | 12 (55%) | 24 (52%) | 48 (71%) | 35 (55%) | 28 (44%) |
|  | Unclear | 18 (24%) | 27 (35%) | 12 (48%) | 6 (27%) | 14 (30%) | 10 (15%) | 19 (30%) | 24 (38%) |
|  | High | 4 (5%) | 10 (13%) | 6 (24%) | 4 (18%) | 8 (17%) | 10 (15%) | 10 (16%) | 12 (19%) |
|  | **Total** | **74 (100%)** | **77 (100%)** | **25 (100%)** | **22 (100%)** | **46 (100%)** | **68 (100%)** | **64 (100%)** | **64 (100%)** |

**Legend**: Data table with data of Figure 2a-h: Development of RoB per time stratum per RoB item.
